# Prevalence, risk factors and impact on outcomes of 30-day unexpected rehospitalization in incident peritoneal dialysis patients

**DOI:** 10.1186/s12882-020-02201-0

**Published:** 2021-01-06

**Authors:** Jianbo Li, Jing Yu, Naya Huang, Hongjian Ye, Dan Wang, Yuan Peng, Xiaobo Guo, Chunyan Yi, Xiao Yang, Xueqing Yu

**Affiliations:** 1 Department of Nephrology, The First Affiliated Hospital, Sun Yat-sen University, Guangzhou, 510080 Guangdong China; 2 Key Laboratory of Nephrology, National Health Commission and Guangdong Province, Guangzhou, 510080 Guangdong China; 3 Department of Statistical Science, School of Mathematics, Sun Yat-sen University, Guangzhou, 510275 Guangdong China; 4 Guangdong Provincial People’s Hospital, Guangzhou, 510080 Guangdong China; 5 School of Medicine, South China University of Technology, Guangzhou, 510080 Guangdong China

**Keywords:** Prevalence, Risk factors, Outcomes, 30-day unexpected rehospitalization, Peritoneal dialysis

## Abstract

**Background:**

Rehospitalization is a major problem for end stage renal disease (ESRD) populations. However, researches on 30-day unexpected rehospitalzation of incident peritoneal dialysis (PD) patients were limited. This study aimed to investigate the prevalence, risk factors and impact on outcomes of 30-day unexpected rehospitalization in incident PD patients.

**Methods:**

This was a retrospective cohort study. Patients who accepted PD catheter implantation in our centre from Jan 1, 2006 to Dec 31, 2013 and regular follow-up were included. The demographic characteristics, laboratory parameters, and rehospitalization data were collected and analyzed. The primary outcome was all-cause mortality, and the secondary outcomes included cardiovascular disease (CVD) mortality and technical failure.

**Results:**

Totally 1632 patients (46.9 ± 15.3 years old, 60.1% male, 25.6% with diabetes) were included. Among them, 149 (9.1%) had a 30-day unexpected rehospitalization after discharge. PD-related peritonitis (*n* = 48, 32.2%), catheter malfunction (*n* = 30, 20.1%) and severe fluid overload (*n* = 19, 12.8%) were the top three causes for the rehospitalization. Multivariate logistic regression analysis showed that length of index hospital stays [Odds ratio (OR) =1.02, 95% confidence interval (CI) 1.00–1.03, *P* = 0.036) and hyponatremia (OR = 1.85, 95% CI 1.06–3.24, *P* = 0.031) were independently associated with the rehospitalization. Multivariate Cox regression analysis indicated that 30-day rehospitalization was an independent risk factor for all-cause mortality [Hazard ratio (HR) =1.52, 95% CI 1.07–2.16, *P* = 0.019) and CVD mortality (HR = 1.73, 95% CI 1.03–2.90, *P* = 0.038).

**Conclusions:**

The prevalence of 30-day unexpected rehospitalization for incident PD patients in our centre was 9.1%. The top three causes for the rehospitalization were PD-related peritonitis, catheter malfunction and severe fluid overload. Thirty-day unexpected rehospitalization increased the risk of all-cause mortality and CVD mortality for PD patients.

## Background

Rehospitalization is a major problem for end stage renal disease (ESRD) populations [1–3]. According to the 2018 United States Renal Data System (USRDS) report, about 35.4% of ESRD patients have an unplanned rehospitalization within 30 days after discharge [1]. Also, rehospitalizations are associated with increased morbidity and mortality and reduced quality of life among dialysis patients [1–3]. Furthermore, inpatient treatment poses a significant financial burden for Medicare expenditures and patients. In 2016, nearly 12 billion dollars had been paid for inpatient care of ESRD patients, accounting for approximately 33% of the total Medicare expenditures for them [1].

Peritoneal dialysis (PD) is a commonly used method of renal replacement therapy for ESRD patients. The readmission rates of PD patients were quite high, 15.5–37.4%, as reported in developed countries [1, 4, 5]. Although several studies have investigated the risk factors and prevention strategies of readmission among hemodialysis (HD) patients [6–9], evidence regarding the prevalence and modifiable risk factors of 30-day readmission among PD patients in developing countries was still limited. In addition, the association of rehospitalization and long-term outcomes among these patients had rarely been described.

In this study, we aimed to investigate the prevalence, causes and risk factors of 30-day unexpected rehospitalization among incident PD patients as well as the association between the rehospitalization and long-term outcomes.

## Methods

### Study design and population

This was a retrospective, single-centre cohort study. Patients who received PD catheter implantation in the Department of Nephrology, the First Affiliated Hospital of Sun Yat-sen University from Jan 1, 2006 to Dec 31, 2013, age ≥ 18 years, and regular follow-up were included. Patients who dropped out during index hospitalization, presenting with a history of malignancy or kidney transplantation, transfer from hemodialysis, or with incomplete data were excluded. Catheter implantation was done with open laparotomy technique by experienced nephrologists following the same procedure [10, 11]. And the implantation was performed by different nephrologists during the eight-year study. All patients were followed until death, withdrawal from PD, or until Aug 31, 2016. The study protocol was approved by the Ethics Committee of The First Affiliated Hospital of Sun Yat-sen University. All participants signed written informed consent forms.

### Demographic and clinical data

Demographic, clinical and laboratory data were collected. Baseline demographic data included age, gender, primary kidney diseases, diabetes mellitus (DM), cardiovascular diseases (CVD) history. CVD was defined as arrhythmias, valvular heart disease, congestive heart failure, angina, myocardial infarction, transient ischemic attack, stroke, or peripheral arterial disease [12]. Clinical data included body mass index (BMI), mean blood pressure, and length of index hospital stay. Baseline laboratory data closest to the discharge date were collected, which included hemoglobin, platelet count, uric acid, corrected calcium, phosphorus, intact parathyroid hormone (iPTH), albumin, total cholesterol (TC), triglyceride (TG), hyponatremia, hypokalemia and creatinine. Corrected calcium was calculated by the conventional Payne equation (c [Ca] mmol/L = t [Ca] + 0.02 × [40 – albumin g/L]) [13]. Hyponatremia was defined as serum sodium level < 135 mmol/L [14]. Hypokalemia was defined as serum potassium level < 3.5 mmol/L [15]. The serum sodium and potassium were measured in the clinical laboratory of our hospital using the indirect ion electrode method (AU5800, Beckman Coulter Inc., Brea, CA, USA).

The main causes for 30-day unexpected rehospitalization were also collected from patients’ files, which were classified as PD-related peritonitis [16], catheter malfunction [11], severe fluid overload (excluding congestive heart failure as it was assigned to CVD) [17], non-peritonitis infection, CVD events [12], abdominal wall hernia [18], refractory hypertension [19] and other causes. The hospitalization in which patients obtained PD catheterization and began PD treatment was defined as the index hospitalization. Thirty-day unexpected rehospitalization was defined as rehospitalization for unexpected clinical events within 30 days after discharge [1]. We excluded emergency visits and scheduled rehospitalizations for routine examination, intravenous iron supplementation, cyclophosphamide pulse therapy, or other planned procedures.

### Outcomes

The primary outcome of this study was all-cause mortality, and the secondary outcomes included CVD mortality and death-censored technical failure. CVD mortality was defined as mortality caused by CVD events [12]. Death-censored technical failure was defined as transfer to HD for more than 90 days from any cause, and it was censored for death, spontaneous recovery of renal function, move to another centre, kidney transplantation, and/or “still on PD” [20] until Aug 31, 2016.

### Statistical analysis

Quantitative variables were displayed as the mean ± standard deviation (SD) for normal distribution and the median (interquartile range, IQR) for skewed distribution. Qualitative variables were expressed as frequencies and percentages. Normally distributed variables were compared using the t-test, and asymmetrically distributed variables were compared using the Wilcoxon rank sum test. Comparisons of categorical variables were tested by the chi-square test. The multivariate logistic regression model was used to identify the independent risk factors that were associated with 30-day unexpected rehospitalization. Survival curves were generated by the Kaplan-Meier method. Multivariate Cox regression models were used to evaluate the association between 30-day unexpected rehospitalization and all-cause mortality, CVD mortality and technical failure. *P* < 0.05 was considered statistical significance. Statistical analyses were performed using SPSS version 19.0 (SPSS Inc., Chicago, IL, USA).

## Results

### Characteristics of the study cohort

**Fig. 1:**
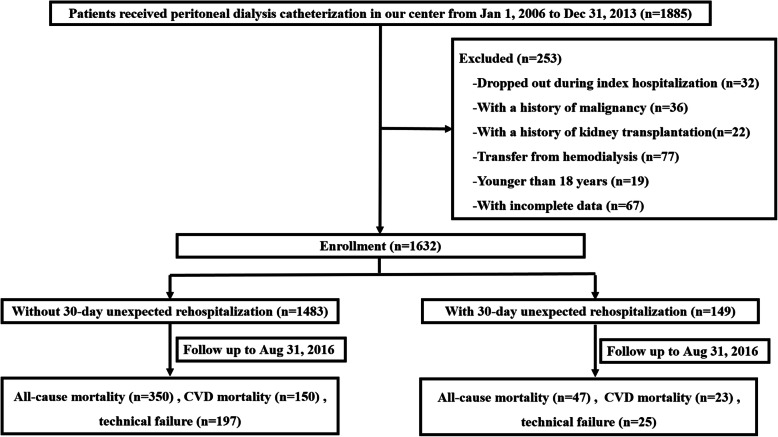
Flow chart for the study participants enrolment and outcomes

**Fig. 2:**
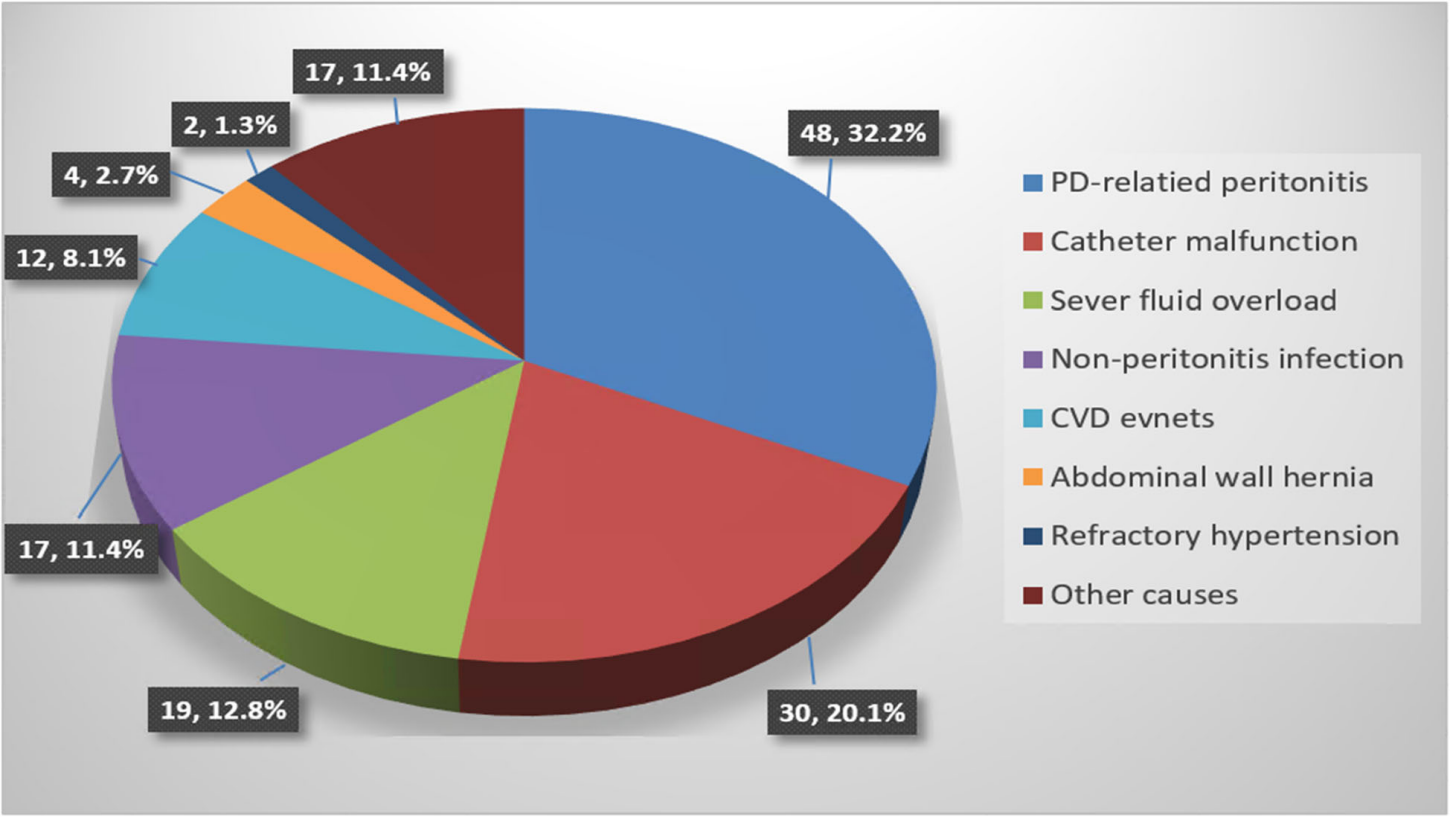
Main causes of 30-day unexpected rehospitalization

A total of 1885 incident PD patients were screened, and finally 1632 patients were included in this study (Fig. 1). The demographic, clinical and laboratory characteristics of the study cohort are shown in Table 1. The average age was 46.9 ± 15.3 years old, males accounted for 60.1% (*n* = 981), and 25.6% (*n* = 417) had DM. Among these patients, 9.1% (*n* = 149) had an unexpected rehospitalization within 30 days after discharge from the index hospitalization, including 2.9% (48/1632) PD-related peritonitis, 1.8% (30/1632) catheter malfunction, 1.2% (19/1632) severe fluid overload, 1.0% (17/1632) non-peritonitis infection, 0.7% CVD events (12/1632), 0.2% (4/1632) abdominal wall hernia, 0.1%(2/1632) refractory hypertension and 1.0% (17/1632) other causes. PD-related peritonitis (48/149, 32.2%), catheter malfunction (30/149, 20.1%) and severe fluid overload (19/149, 12.8%) were the top three causes for rehospitalization (Fig. 2). Among the 48 peritonitis-related rehospitalizations, 22 (45.8%) were culture negative, 2(4.2%) had no data, 8(16.7%) were caused by *Escherichia coli*, 4(8.3%) were *Staphylococcus aureus*, 3 were *Streptococcus,* another 3 were *Staphylococcus epidermidis*, 2 were *Klebsiella pneumoniae*, the rest 4 were other bacteria. And among these peritonitis episodes, 11(22.9%) were related to patient’s techniques, which defined as unqualified operating environment or unqualified operating methods. Fifteen episodes of catheter malfunction were caused by omental wrap which resolved by omental release surgery, 11 were caused by functional catheter shift which resolved by conservative treatment, 2 were caused by catheter shift which resolved by catheter reposition surgery, and 2 were caused by other reasons.

**Table 1:** Comparison of baseline parameters between patients without (group 1) and with (group 2) 30-day unexpected rehospitalization

Variables	Total (*n* = 1632)	Group 1(*n* = 1483)	Group 2 (*n* = 149)	*P* value
Age (years)	46.9 ± 15.3	46.6 ± 15.2	50.2 ± 16.8	**0.013**
Gender (male) n (%)	981 (60.1)	892 (60.1)	89 (59.7)	0.921
Primary kidney diseases n (%)				0.331
Chronic glomerulonephritis	991 (60.7)	905 (61.1)	85 (57.0)	–
Diabetic nephropathy	365 (22.4)	324 (21.8)	41 (27.5)	–
Hypertensive nephropathy	113 (6.9)	106 (7.1)	7 (4.7)	–
Others	163 (10.0)	147 (9.9)	16 (10.7)	–
Diabetes mellitus n (%)	417 (25.6)	371 (25.0)	46 (30.9)	0.118
CVD history n (%)	348 (21.3)	313 (21.1)	35 (23.5)	0.498
BMI (kg/m^2^)	21.8 ± 3.2	21.8 ± 3.2	22.0 ± 3.2	0.372
Mean blood pressure (mmHg)	110.4 ± 16.8	109.0 ± 16.7	110.0 ± 18.5	0.760
Length of index hospital stay (day)^a^	23.7 ± 10.1	23.5 ± 9.9	26.3 ± 12.0	**0.006**
Hemoglobin (g/L)	79.2 ± 18.7	79.2 ± 18.7	78.3 ± 18.1	0.539
Platelet (10^9^/L)	193.6 ± 78.7	193.9 ± 79.2	190.6 ± 73.4	0.627
Uric acid (μmol/L)	488.5 ± 149.5	490.2 ± 148.7	471.7 ± 157.0	0.161
Corrected calcium (mmol/L)	2.1 ± 0.3	2.1 ± 0.3	2.1 ± 0.3	0.290
Phosphorus (mmol/L)	2.0 ± 0.6	2.1 ± 0.6	2.0 ± 0.5	0.196
iPTH (pg/mL)	348.7 (201.7540.2)	351.8 (204.7539.0)	333.9 (175.2551.2)	0.521
Albumin (g/L)	34.7 ± 5.1	34.8 ± 5.1	33.8 ± 5.7	**0.016**
TC (mmol/L)	4.7 ± 1.4	4.7 ± 1.4	4.7 ± 1.6	0.802
TG (mmol/L)	1.3 (0.9,1.9)	1.3 (0.9,1.9)	1.3 (1.0,2.0)	0.664
Hyponatremia n (%)	105 (6.9)	87 (6.3)	18 (12.9)	**0.003**
Hypokalemia n (%)	364 (23.8)	328 (23.6)	36 (25.7)	0.578
Creatinine (μmol/L)	793.7 ± 286.9	794.8 ± 283.6	782.1 ± 318.9	0.648

Values were expressed as mean ± SD, median (interquartile range), or number (percentage)

*Abbreviations*: *CVD* cardiovascular disease, *BMI* body mass index, *iPTH* intact parathyroid hormone, *TC* total cholesterol, *TG* triglyceride

^a^Length of index hospital stay refers to the hospitalization days during which patients obtained peritoneal dialysis (PD) catheterization and began PD treatment

Compared with those who did not have 30-day unexpected rehospitalization (group 1), patients in the rehospitalization group (group 2) were older [50.2 ± 16.8 years verse (vs.) 46.6 ± 15.2 years, *P* = 0.013)], had longer length of index hospital stay (26.3 ± 12.0 days vs. 23.5 ± 9.9 days, *P* = 0.006), lower level of albumin (33.8 ± 5.7 g/L vs. 34.8 ± 5.1 g/L, *P* = 0.016), higher proportion of hyponatremia [12.9% vs. 6.3%, *P* = 0.003]. However, there were no significant differences in gender, causes of primary kidney diseases, DM, CVD history, BMI, mean blood pressure level, and levels of hemoglobin, platelet, uric acid, corrected calcium, phosphorus, iPTH, TC, TG, creatinine, and proportion of hypokalemia between the two groups.

### Risk factors associated with unexpected 30-day rehospitalization

Univariate logistic regression analysis revealed that advanced age, longer length of index hospital stay and hyponatremia were positively correlated with the rehospitalization, and albumin level was negatively correlated with the rehospitalization (Table 2). Multivariate logistic regression analysis showed that length of index hospital stay [Odds ratio (OR) = 1.02, 95% confidence interval (CI) 1.00–1.03, *P* = 0.036] and hyponatremia (OR = 1.85, 95% CI 1.06–3.24, *P* = 0.031) were positively independently associated with 30-day unexpected rehospitalization after adjusting for age, gender, diabetes mellitus, CVD history and albumin (Table 2).

**Table 2:** Factors associated with 30-day unexpected rehospitalization in the logistic regression model

Variables	Univariate		Multivariate	
	OR(95%CI)	*P* value	OR(95%CI)	*P* value
Age (every 1 year↑)	1.02 (1.00,1.03)	**0.007**	1.01 (1.00,1.03)	0.091
Gender (female vs. *male*)	1.02 (0.72,1.44)	0.921	1.08 (0.75,1.54)	0.691
Diabetes mellitus (yes vs.no)	1.34 (0.93,1.93)	0.119	1.08 (0.70,1.67)	0.738
CVD history (yes vs.no)	1.15 (0.77,1.71)	0.499	0.90 (0.57,1.41)	0.633
Length of index hospital stay (every 1 day↑)^a^	1.02 (1.00,1.04)	**0.001**	**1.02 (1.00,1.03)**	**0.036**
Hyponatremia (yes vs.no)	2.21 (1.29,3.79)	**0.004**	**1.85 (1.06,3.24)**	**0.031**
Albumin (every 1 g/L↑)	0.96 (0.93,0.99)	**0.017**	0.97 (0.94,1.01)	0.104

Variables that *p* < 0.1 in univariate analysis and with clinical significance were included in multivariate analysis

*Abbreviations*: *OR* odds ratio, *CI* confidence interval, *vs.* verse, *CVD* cardiovascular disease

^a^Length of index hospital stay refers to the hospitalization days during which patients obtained peritoneal dialysis (PD) catheterization and began PD treatment

### Association of unexpected 30-day rehospitalization with clinical outcomes

**Fig. 3:**
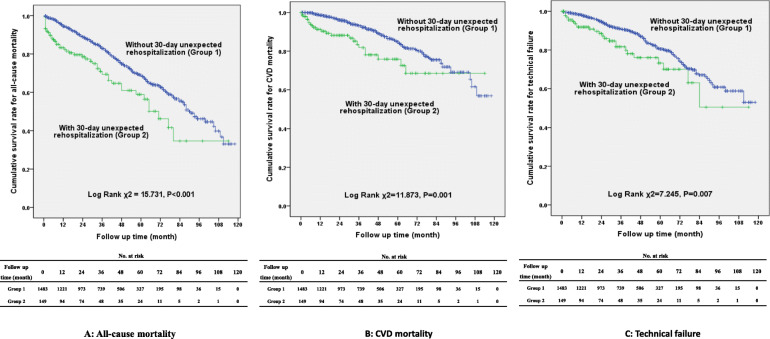
Survival curves for patients without and with 30-day unexpected rehospitalization

After a median of 34.5 (15.0, 55.0) months’ follow-up, 397 (24.3%) patients died, of which 173 (10.6%) were caused by CVD, and 222 (13.6%) patients had technical failure. Patients in the rehospitalization group had higher rates of all-cause mortality (31.5% vs. 23.6%*, P* = 0.031) and CVD mortality (15.4% vs. 10.1%, *P* = 0.044) compared with the non-rehospitalization group. The proportion of technical failure between the two groups was comparable (16.8% vs. 13.3%, *P* = 0.236). Survival curves showed that the cumulative incidence of all-cause mortality (Log Rank χ^2^ = 15.731, *P* < 0.001), CVD mortality (Log Rank χ^2^ = 11.873, *P* = 0.001) and technical failure (Log Rank χ^2^ = 7.245, *P* = 0.007) were significantly higher in the rehospitalization group compared with the non-rehospitalization group (Fig. 3). Multivariate Cox regression analysis showed that 30-day unexpected rehospitalization was an independent risk factor for all-cause mortality [Hazard ratio (HR) =1.52, 95% CI 1.07–2.16, *P* = 0.019) after adjusting for age, gender, diabetes mellitus, CVD history, BMI, length of index hospital stay, hemoglobin, platelet, corrected calcium, phosphorus, iPTH, albumin, TC, TG, hyponatremia, hypokalemia, uric acid and creatinine, and for CVD mortality (HR = 1.73, 95% CI 1.03–2.90, *P* = 0.038) after adjusting for age, gender, diabetes mellitus, CVD history, BMI, length of index hospital stay, hemoglobin, platelet, corrected calcium, phosphorus, iPTH, albumin, TC, TG, uric acid and creatinine (Table 3).

**Table 3:** Association between 30-day unexpected rehospitalization and outcomes in Cox regression models

Outcomes	HR (95%CI)	*P* value
All-cause mortality		
Unadjusted	1.83 (1.35,2.49)	**< 0.001**
Adjusted^a^	1.52 (1.07,2.16)	**0.019**
CVD mortality		
Unadjusted	2.12 (1.37,3.30)	**0.001**
Adjusted^b^	1.73 (1.03,2.90)	**0.038**
Technical failure		
Unadjusted	1.76 (1.16,2.67)	**0.008**
Adjusted^c^	1.57 (1.00,2.48)	**0.052**

Variables that *p* < 0.1 in univariate analysis and with clinical significance were included in multivariate analysis

*Abbreviations*: *HR* hazard ratio, *CI* confidence interval, *CVD* cardiovascular disease, *BMI* body mass index, *iPTH* intact parathyroid hormone, *TC* total cholesterol, *TG* triglyceride

^a^Variables including age, gender, diabetes mellitus, CVD history, BMI, length of index hospital stay, hemoglobin, platelet, corrected calcium, phosphorus, iPTH, albumin, TC, TG, hyponatremia, hypokalemia, uric acid, creatinine were adjusted

^b^Variables including age, gender, diabetes mellitus, CVD history, BMI, length of index hospital stay, hemoglobin, platelet, corrected calcium, phosphorus, iPTH, albumin, TC, TG, uric acid, creatinine were adjusted

^c^Variables including age, gender, diabetes mellitus, CVD history, BMI, albumin, TG, hypokalemia were adjusted

## Discussion

In the present study, we found that the prevalence of 30-day unexpected rehospitalization in incident PD patients was 9.1% in our PD centre. The top three causes for the rehospitalization were PD-related peritonitis, catheter malfunction and severe fluid overload. Length of index hospital stay and hyponatremia were independently associated with 30-day unexpected rehospitalization. Furthermore, 30-day unexpected rehospitalization increased the risk of all-cause mortality and CVD mortality for incident PD patients.

Previous studies reported that rehospitalization rates were 15.8–37.5% in HD patients [1, 4, 6, 7, 9] and 15.5–37.4% in PD patients [1, 4, 5]. Compared to previous studies, the prevalence of 30-day unexpected rehospitalization in our study was much lower. One of the probable explanations might be that patients in our cohort were much younger than those in previous studies. In our study, the mean age was 46.9 ± 15.3 years old, while in the aforementioned studies, the mean age range was 57–66 years old. Older age was a risk factor for morbidity and mortality in the incident dialysis patients [21], and was also found to be an independent risk factor for unexpected rehospitalization for PD patients [5]. Secondly, the comorbid status of DM in our cohort was much less than that in the other studies. The proportion of diabetes in our patients was 25.6%, while in Li Z’s study, the proportion was 42.1% [5]. And in Ziv Harel’s study, the proportion of patients with DM was as high as 62.0% [7]. DM is strongly associated with macro- and microvascular complications, including CVD, retinopathy, nephropathy, and neuropathy [22]. These complications might increase the risk of readmission. Thirdly, patients in our study were incident patients, while most of the previous studies included prevalent patients who had pretty long dialysis periods with more comorbidities and worse status [1–4, 6, 7, 9]. Moreover, the specific follow-up management strategies of our centre might also be attributable to the lower rate of unexpected rehospitalization [23–25]. In our center, patients were followed up 3 times within 1 month after discharged. The first follow-up was done by phone within 3 days to make sure that patients were familiar with PD operation and took medications as directed. The second follow-up was done by phone within 2 weeks to evaluate whether their dialysis prescriptions were appropriate, especially whether there was fluid overload. The third follow-up was done within 3–4 weeks by clinical visit to assess the comprehensive health condition after PD initiation. The patients could come back to visit their nephrologist through green channel, and a 24-h on-call service was always provided in the PD center to deal with their emergent problems. Additionally, as most of our patients were living in remote rural areas, we established a PD “satellite center” program across Guangdong Province to provide standardized training for doctors and nurses in satellite hospitals who provided cares to patients in remote areas [25]. The PD patients who had clinical symptoms in the early stage could be interventedtimely, which would prevent them from deteriorating to the point of admission [23]. All of the above factors may contribute to the decreased rate of the unexpected rehospitalization in our cohort.

We identified that length of index hospital stay was an independent risk factor of 30-day unexpected rehospitalization. Patients with longer hospital stay always presented with more severe or complicated disease condition during index admission [26]. Such complex condition not only prolonged their index hospital care, but also made them more vulnerable to an unexpected rehospitalization. Thus post-discharge care plan for these patients should be made carefully. Additionally, we revealed that hyponatremia was another risk factor of rehospitalization. It was reported that hyponatremia was associated with increased risk of 30-day rehospitalization among patients with congestive heart failure [27]. While in PD patients, hyponatraemia was always accompanied with hypokalaemia [28], which was a well-recognized risk parameter for peritonitis [29, 30]. In addition, hyponatremia was reported to be a surrogate marker of longer hospital stay and poorer outcome for PD-related peritonitis [31]. Also, hyponatremia was reported to be related to a lower level of albumin [32], which would significantly increase the risk of peritonitis and other infectious diseases [33]. Moreover, hyponatremia always resulted from inappropriate water gain among PD patients [32, 34]. Persistent water retention would lead to severe fluid overload or refractory hypertension and finally cause rehospitalization. In addition, it was demonstrated that hyponatremia was strongly correlated with the decline of residual renal function (RRF) [35], which is a well-recognized risk factor of fluid overload [17]. In particular, hyponatremia has been found to be significantly associated with an increased risk of infection-related hospitalization and new-onset CVD events for dialysis patients [36, 37]. All of the above factors increased the risk of rehospitalization for incident PD patients.

It has been reported that rehospitalizations were associated with increased morbidity and mortality and reduced quality of life among dialysis population [1–3]. In the current study, we revealed that 30-day unexpected rehospitalization was independently associated with poor long-term outcomes of incident PD patients. A probable explanation for this finding is that the adverse clinical events that cause early unexpected rehospitalization might also lead to worse prognosis. First, peritonitis was the most common cause of rehospitalization in our study. It is well known that peritonitis could lead to the failure of peritoneal function, resulting in transferring to HD or even death [16, 38]. Our previous researches demonstrated that early onset peritonitis in incident PD patients affected not only the peritoneal function but also the confidence and compliance of the patients in the treatment modality, which in turn led to worse outcomes [33]. Second, catheter malfunction was the other important cause of rehospitalization, accounting for 1.8% of our study population, which also was an important cause of early technical failure of PD. Although the prevalence of catheter malfunction was relatively low in our centre, it was still an important cause of 30-day unexpected rehospitalization. Peritonitis and catheter failure were the most common causes of readmission which related to patient education and experience of surgeon. Previous studies by our colleagues have shown that lower education level is associated with the first episode of peritonitis [29] and long-term all-cause mortality [39]. We also found that severe fluid overload was another important reason for rehospitalization in this cohort, accounting for 1.2% of our study population. Fluid overload not only played an inverse role in the preservation of RRF [17, 40] but also increased both all-cause and CVD mortality [17]. All of these aforementioned events could lead to poor outcomes. The rest of the rehospitalization reasons, such as CVD events, non-peritonitis infection, etc., would also result in a poor prognosis undoubtedly [3, 20].

Our study has several limitations. First, all of the data were collected from a single centre. The results may not be generalizable to other centres. Second, we were not able to consider all variables potentially associated with the rehospitalization and long-term outcomes. Further studies that take into account more risk factors, such as post-discharge medical care, health literacy and social support, are warranted. Nonetheless, to our knowledge, this was the first study concerning 30-day unexpected rehospitalization in a large cohort of incident PD patients in developing country. The strengths of our study included its large cohort and complete follow-up data. The results of our study might be of value in guiding clinical practice.

## Conclusions

In summary, the prevalence of 30-day unexpected rehospitalization for incident PD patients in our centre was 9.1%. The top three causes for the rehospitalization were PD-related peritonitis, catheter malfunction and severe fluid overload. Length of index hospital stay and hyponatremia were independently associated with the rehospitalization. Thirty-day unexpected rehospitalization increased the risk of all-cause mortality and CVD mortality for PD patients.

## Data Availability

The data used in the current study are available from the corresponding author on reasonable request.

## Abbreviations

ESRD: End stage renal disease
PD: Peritoneal dialysis
CVD: Cardiovascular disease
OR: Odds ratio
CI: Confidence interval
HR: Hazard ratio
USRDS: United States Renal Data System
HD: Hemodialysis
DM: Diabetes mellitus
BMI: Body mass index
iPTH: Intact parathyroid hormone
TC: Total cholesterol
TG: Triglyceride
SD: Standard deviation
IQR: Interquartile range
RRF: Residual renal function
